# Supplementary material for: Content-rich biological network constructed by mining PubMed abstracts
Source: BMC Bioinformatics. 2004 Oct 8;5:147. doi: 10.1186/1471-2105-5-147 (PMC528731; doi:10.1186/1471-2105-5-147)
Supplement: Additional File 2 — The original results of the above study (non-essential files are deleted to keep the file size under the limit set by BMC bioinformatics). [file 1471-2105-5-147-S2.bz2 › chilibotAdditionalFile2/dip10/568ID8386828E10932/html/FGF5_FGFR1.html]

 


 **FGF5** and **FGFR1** 
  
Found 20 abstracts in PubMed, retrieved 10.  
 

 What does Google say? 
 PDF only 
| .edu only 

---

**Interactive relationship** (e.g. stimulation, inhibition, etc)

**Stimulatory relationship**- In contrast, TAKA 1 clones expressing FGFR 1  [ **FGFR1** ]  IIIc were growth stimulated in the presence of FGF 5  [ **FGF5** ]  and displayed enhanced mitogen activated protein kinase MAPK activity in the presence of FGF 5  [ **FGF5** ] .  Ref: 11876253 Int J Pancreatol, 2001
**Neutral relationship**- FGF 5  [ **FGF5** ]  is a secreted FGF that is believed to preferentially act via the IIIc splice variant of FGFR 1  [ **FGFR1** ] .  Ref: 11876253 Int J Pancreatol, 2001

**Non-interactive relationship** (e.g. studied together, co-existance, homology, etc.)

- AIM In this study we investigated the importance of FGFR 1  [ **FGFR1** ]  IIIc expression for FGF 5  [ **FGF5** ]  mitogenic signaling in a pancreatic ductal cell line.  Ref: 11876253 Int J Pancreatol, 2001
- CONCLUSION Our data demonstrate that FGFR 1  [ **FGFR1** ]  IIIc can mediate FGF 5  [ **FGF5** ]  induced mitogenesis via the MAPK pathway in pancreatic ductal cells, and suggest that expression of FGFR 1  [ **FGFR1** ]  IIIc in conjunction with FGF 5  [ **FGF5** ]  may contribute to the pathobiology of human pancreatic cancer.  Ref: 11876253 Int J Pancreatol, 2001
- A cDNA encoding FGFR 1  [ **FGFR1** ]  IIIc was stably transfected into the well differentiated TAKA 1 pancreatic ductal cell line that is not responsive to  **FGF5**  and does not express FGFR 1  [ **FGFR1** ] .  Ref: 12105858 Gastroenterology, 2002
- RESULTS We detected the expression of mRNAs for FGF1 through  **FGF5** , FGF7 through FGF10, and  **FGFR1**  through FGFR4 IIIb and IIIc splice variants in rat metanephroi from E14 to E21.  Ref: 10594778 Kidney Int, 1999
- Given that FGF 5  [ **FGF5** ]  receptor  **FGFR1**  is expressed in dermal papilla cells DPCs, which are known to stimulate outer root sheath cell ORSC proliferation, we hypothesized that FGF 5  [ **FGF5** ]  attenuates DPC mediated ORSC proliferation.  Ref: 11779149 Biochem Biophys Res Commun, 2002
- RESULTS TAKA 1 cells secrete FGF 5  [ **FGF5** ] , but were found not to express FGFR 1  [ **FGFR1** ]  and to be unresponsive to exogenous FGF 5  [ **FGF5** ] .  Ref: 11876253 Int J Pancreatol, 2001
- Human pancreatic ductal carcinoma cells express FGF 5  [ **FGF5** ]  and FGFR  [ **FGFR1** ]  1IIIc, implying a potential for autocrine growth modulation.  Ref: 11876253 Int J Pancreatol, 2001
